# Determinants of Tetanus Vaccination among Adult Immigrants: Findings from the Portuguese National Health Survey 2014

**DOI:** 10.3390/ijerph16091619

**Published:** 2019-05-09

**Authors:** Sofia Moura, Maria do Rosário O. Martins

**Affiliations:** 1 Global Health and Tropical Medicine, GHTM, Instituto de Higiene e Medicina Tropical, IHMT, Universidade Nova de Lisboa, UNL, Rua da Junqueira 100, 1349-008 Lisbon, Portugal; mrfom@ihmt.unl.pt; 2 Global Public Health Doctoral Programme, Instituto de Higiene e Medicina Tropical, IHMT, Universidade Nova de Lisboa, UNL, Rua da Junqueira 100, 1349-008 Lisbon, Portugal

**Keywords:** tetanus, vaccination, immigrants, Portugal

## Abstract

Vaccination is an effective strategy to prevent tetanus, and in Portugal this service is provided free of charge. Despite this, immigrants reported lower tetanus vaccination coverage than did Portuguese natives. The objective of this study was to identify sociodemographic, migration-related, and access-to-care factors associated with tetanus vaccination coverage among adult immigrants, using data from the Portuguese National Health Survey 2014. For the sample of 1277 immigrants aged ≥18 years, we estimated self-reported tetanus vaccination within the preceding 10 years and its determinants using complex samples logistic regression. The overall self-reported tetanus vaccination coverage was 79.5% (95% CI: 75.8–82.8). Age (adjusted odd ratio (aOR) per 1 year age increase = 0.97, 95% CI: 0.95–0.99), higher household income per adult (aOR = 0.42, 95% CI: 0.19–0.96), having Portuguese citizenship (aOR = 2.30, 95% CI: 1.25–4.24), having private health insurance (aOR = 1.99, 95% CI: 1.06–3.71), and contact with family/general physician in the last 12 months (aOR = 1.59, 95% CI: 1.01–2.51) were associated with self-reported tetanus vaccination coverage among adult immigrants. We also found significant disparities in coverage between regions of residence. This study identified several determinants associated with self-reported tetanus vaccination coverage among adult immigrants in Portugal. These findings may help policymakers to design specific interventions to increase tetanus vaccination coverage among this population.

## 1. Introduction

Widespread vaccination has greatly reduced the burden of tetanus, which is a potentially fatal disease. In countries with a well-established immunization program, tetanus is now a rare disease as a result of high vaccination coverage. Still, in several parts of the world this disease remains a health problem where cases tend to occur in individuals without immunity against tetanus due to a lack of vaccination or waning immunity [1,2].

In Portugal, a tetanus-toxoid-containing vaccine was introduced in the national immunization program (NIP) in 1965; and the last case of neonatal tetanus was reported in 1997 [3]. In 2001, the tetanus and diphtheria toxoid (Td) vaccine replaced the tetanus toxoid vaccine as the decennial booster dose in adulthood [4]. In 2017, the NIP schedule was updated with longer intervals between booster doses with adults receiving Td boosters at 25, 45, and 65 years of age, followed by a dose every 10 years [5]. As protection against tetanus does not benefit from herd immunity, it is necessary that all adults receive timely vaccination with the recommended number of doses in order to maintain protective immunity throughout life [6]. The Portuguese NIP is universal and free of charge for those living in Portugal [4], including adult migrants regardless of their immigration status [7]. Nonetheless, immigrants can still have difficulties accessing this preventive health service as a result of cultural, linguistic, and geographical barriers [8,9]. In addition, administrative procedures and provider’s attitudes can also pose a barrier to access [10]. In fact, a study conducted in Portugal using data from the National Health Survey 2014 (NHS 2014) identified significant disparities for self-reported tetanus vaccination coverage between immigrants and Portuguese natives. In this study, immigrants without Portuguese nationality reported lower vaccination coverage (68.8%) as compared to the Portuguese-born population (83.5%) [11]. A significant difference can also be seen when comparing adult immigrants with natives in Portugal, as shown in the Supplementary Table S1. Those born outside Portugal reported a significantly lower tetanus vaccination coverage (79.5%) when compared to those born in Portugal (83.0%).

The overall goal of an immunization program is to prevent disease and consequently promote patient and public health [12]. Therefore, it is necessary to identify factors related to its underuse and develop strategies to increase vaccine uptake in adults [13], considering potentially vulnerable groups, such as immigrants [2]. To our knowledge, no current literature exists regarding identification of factors associated with tetanus vaccination among adult immigrants in Portugal. Furthermore, comprehensive information regarding other vaccination uptake in this population is also limited.

In order to address this gap, the goal of this study was to identify sociodemographic, migration-related, and access-to-care factors associated with tetanus vaccination coverage among adult immigrants in Portugal, by examining data from NHS 2014.

## 2. Materials and Methods

### 2.1. Study Design and Population

The NHS 2014 was conducted by Statistics Portugal in collaboration with the National Health Institute Doutor Ricardo Jorge. This survey is a population-based cross-sectional study with a sample of 22,538 households, obtained through multistage and cluster sampling; 18,204 persons living in Portugal (≥15 years of age) were surveyed and information on health status, healthcare, health determinants, and sociodemographic characteristics of individuals was collected between September and December 2014. Overall, the interview response rate was 80.8%. Details regarding the NHS methodology are described elsewhere [14].

In this study, we work with the results of the NHS 2014 related to adult immigrants aged 18 years or older (n = 1304). We excluded 27 participants with data missing on the determinants of interest. Therefore, our study was based on a sample of 1277 adult immigrants.

### 2.2. Measures

The outcome variable was self-reported tetanus vaccination coverage (vaccination within the preceding 10 years), which was assessed by two questions, “Have you ever been vaccinated against tetanus?” and, if yes, “When was your last tetanus shot? Less than ten years or ten years or more?”

Migrant status was defined according to country of birth. As for migration-related factors, we included in the analysis region of birth, length of residence in Portugal (in years), and citizenship. Immigrants were grouped into two regions of birth: European Union (EU) member states and non-EU member states. Regarding citizenship, immigrants were classified as Portuguese citizens and not Portuguese citizens. Length of residence in Portugal was used as a continuous variable. Sociodemographic factors included gender, age (in years), marital status, educational level, employment status, household income per adult, degree of urbanization, region of residence, and self-reported health status. Regarding access-to-care factors, we used information about private health insurance, public healthcare, and last contact with family/general physician.

### 2.3. Statistical Analysis

We used descriptive statistics to characterize adult immigrants (≥18 years of age) living in Portugal. We estimated self-reported tetanus vaccination coverage (within the preceding 10 years) and the corresponding 95% confidence intervals (CI). To compute adjusted odds ratios (aOR) and their respective 95% CI, and to assess the determinants of self-reported tetanus vaccination coverage among adult immigrants in Portugal, we used a multivariable complex samples logistic regression approach. All analyses were carried out with IBM^®^ SPSS^®^ Statistics 25 complex samples module (IBM Corp., Armonk, NY, USA), which incorporates strata, primary sampling units, and weight variables. We used a significance level of 5%.

## 3. Results

Data include a sample of 1277 adult immigrants living in Portugal in 2014. Table 1 presents the sociodemographic, migration-related, and access-to-care characteristics of participants. Adult immigrants had a mean age of 44 ± 14 years, ranging from 18 to 95 years. The majority were female (54.1%), married or living with their partner (67.0%), and employed (61.4%). Most lived in the *Lisboa* region (44.1%), where the capital city is located. The majority of participants were born in non-EU member states (75.6%) and were Portuguese citizens (74.7%). The median length of residence in Portugal was 26 years (ranging from less than 1 year to 85 years). Only 24.6% of participants had private health insurance.

The overall self-reported tetanus vaccination coverage (vaccination in the past 10 years) among adult immigrants was 79.5% (95% CI: 75.8–82.8). A lower tetanus vaccination coverage (58.1%, 95% CI: 45.0–70.1) was reported by older immigrants (≥65 years of age).

In addition to self-reported vaccination coverage, we also calculated the percentage of missed opportunities for tetanus vaccination among adult immigrants. Of those without a tetanus shot within the previous 10 years, 66.3% had visited a family/general physician in the past 12 months, and 1.9% had an injury-related hospital visit in the past 12 months.

Table 2 provides the results of aOR for sociodemographic, migration-related, and access-to-care factors, and self-reported tetanus vaccination coverage. The logistic regression analysis of age on self-reported tetanus vaccination coverage resulted in an aOR per 1 year of age increase of 0.97 (95% CI: 0.95–0.99). Factors associated with increased odds of self-reported tetanus vaccination coverage were having Portuguese citizenship (aOR = 2.30, 95% CI: 1.25–4.24), having private health insurance (aOR = 1.99, 95% CI: 1.06–3.71), and contact with family/general physician in the last 12 months (aOR = 1.59, 95% CI: 1.01–2.51). Adult immigrants living in the *Lisboa*, *Algarve*, and *Região Autónoma (RA) da Madeira* regions were less likely to report tetanus vaccination when compared to those living in the *Norte* region (aOR = 0.28, 95% CI: 0.12–0.64; aOR = 0.31, 95% CI: 0.11–0.82; aOR = 0.30, 95% CI: 0.11–0.77, respectively). Having a higher household income (quintile 5) decreased the odds of self-reported tetanus vaccination coverage when compared with those with the lowest income (quintile 1) (aOR = 0.42, 95% CI: 0.19–0.96).

## 4. Discussion

Tetanus is not a communicable disease, and therefore protection against this disease does not benefit from herd immunity. Hence, an individual who is not adequately vaccinated may be at risk of developing tetanus [6]. In our study, the overall percentage of adult immigrants who reported receiving a tetanus booster dose within the preceding 10 years was 79.5% (95% CI: 75.8–82.8). This vaccination coverage is below the ultimate goal of achieving 100% [3], but is higher than the self-reported tetanus vaccination coverage among immigrants found in other countries, which ranges from 17.0% to 65.2% [9,13,15,16,17,18].

Vaccination coverage is an indicator used to measure the performance of immunization programs [19]. In light of the inequalities in self-reported tetanus vaccination coverage between immigrants and Portuguese natives found by Shaaban et al. [11], we sought to identify sociodemographic, migration-related, and access-to-care factors associated with tetanus vaccine uptake among adult immigrants. Our findings showed that age, household income per adult, region of residence, citizenship, private health insurance, and contact with family/general physician are associated with self-reported tetanus vaccination coverage among adult immigrants.

In our study, we observed that self-reported tetanus vaccination coverage decreased with age, with older immigrants (≥65 years of age) reporting a tetanus vaccination coverage of only 58.1% (95% CI: 45.0–70.1). This finding is in line with other studies conducted among immigrants [9,18]. As this decrease with age was also observed among Portuguese natives (data not shown), it would be beneficial to carry out a vaccination catch-up campaign targeting older age groups, regardless of an individuals’ immigration status.

Adult immigrants with the highest household income per adult were less likely to report tetanus vaccination when compared to those with the lowest income. This finding is contrary to other studies conducted among adult immigrants [18], and in the general adult population [13]. However, this is in line with our belief that financial barriers are not a factor associated with vaccination uptake because vaccines included in the Portuguese NIP are free of charge. Additionally, a few studies [20,21] found lower vaccination rates in high-income areas. In view of these results, further research with focus on knowledge, attitudes, and beliefs of individuals from high-income families toward vaccination may be useful to better understand this association.

We found significant differences in self-reported tetanus vaccination coverage by region of residence. Adult immigrants living in the *Algarve*, *Lisboa*, and *RA da Madeira* regions reported the lowest coverages, while those living in the *Centro* and *Norte* regions reported the highest ones, with coverages greater than 90%. According to estimates from Statistics Portugal, in 2014 the *Lisboa*, *Algarve*, and *RA da Madeira* regions had the lowest tetanus vaccination coverages among older children (14 years of age) in the general population [22]. These results are congruent with the results found in our study, which may indicate different implementation practices among regions.

Of the migration-related factors, only citizenship significantly affected the odds of reporting tetanus vaccination. Adult immigrants with Portuguese citizenship reported significantly higher vaccination coverage than those without it. Sánchez-González et al. [18] found a different result in a study conducted among adult immigrants in the United States. In their study, none of the migration-related factors examined (country of birth, citizenship, language of the interview, and length of residence) were associated with the outcome, suggesting that all foreign-born residents would benefit from similar strategies to address barriers to accessing this type of preventive health service. The discrepant findings between studies may be due to differences in the healthcare systems between Portugal and the United States, which may translate into different barriers in healthcare service access among immigrants. In our study, we do not have information regarding how individuals born outside Portugal obtained Portuguese citizenship. This data may be important to explore in future studies to understand how Portuguese citizenship among immigrants leads to increased access to tetanus vaccination.

Of the access-to-care factors, private health insurance and contact with family/general physician significantly affected the odds of reporting tetanus vaccination. Adult immigrants with private health insurance reported significantly higher vaccination coverage than those without it. This finding is in line with a study conducted in the United States [18], and similar findings have also been reported for the overall Portuguese adult population regarding other preventive health services [23,24]. Tetanus vaccination is part of the Portuguese NIP, which is universal and free of charge for those living in Portugal [4], including adult migrants [7]. For this reason, having private health insurance should not have interfered with tetanus vaccine uptake. As perceived need and perceived value were described as predictors of private health insurance coverage [25,26], a possible explanation for our finding is that adult immigrants with private health insurance are more concerned with their health status, and therefore more aware of preventive services, such as vaccination. Another possible explanation is that, since vaccines included in the NIP can also be administered at some private healthcare centers, immigrants with private health insurance could more easily overcome barriers to accessing public healthcare services.

Adult immigrants that visited a family/general physician in the past 12 months reported significantly higher vaccination coverage than those not reporting such a visit. This finding is in line with studies conducted among adult immigrants [18], and in the general adult population [13]. This result highlights the importance of such visits and the crucial role of health professionals in increasing tetanus vaccination uptake. However, 66.3% of adult immigrants without a tetanus shot within the previous 10 years had visited a family/general physician in the past 12 months. As tetanus vaccination in Portugal is mandatory [27], we are inclined to classify this as a missed opportunity for vaccination rather than as a personal and conscious choice of the participant to not receive the vaccine.

When analyzing tetanus vaccination coverage, an important aspect to consider is that not having received a tetanus booster dose in the previous 10 years does not necessarily mean a lack of immunity to tetanus. Several studies have shown that vaccine-induced immunity to tetanus can last more than 10 years [28,29,30,31]. The alteration made to the Portuguese NIP in 2017, replacing the decennial adult booster doses by longer intervals [5], is in line with this finding. In view of this alteration, now more than ever it is important to comply with the NIP schedule, because the intervals to receive a booster dose are longer, and missing one dose may represent a potentially higher risk of developing tetanus. The problem of not receiving timely vaccination is more evident when immigrants come from countries with less developed tetanus immunization programs, which have low primary immunization coverage, or do not have adult booster doses [2]. In 2014, most of the 10 major foreign communities in Portugal belonged to these countries, and in 2017 the situation was similar.

As pointed out by the World Health Organization, health systems should consider social determinants of health when improving the healthcare services available to migrants and ethnic minorities [32]. To the best of our knowledge, this is the first study conducted in Portugal to identify determinants of tetanus vaccination among adult immigrants. The findings in this study, together with information about barriers to access to healthcare services described by other authors [10,33,34], may help policymakers develop strategies and interventions to improve tetanus vaccination coverage among immigrants in Portugal.

The main limitations of this study are the following. First, the NHS 2014 was designed to be representative of the population living in Portugal, aged 15 years or over, but not of the adult immigrant population. Therefore, the sample of immigrants included in this study may not be representative of all immigrants. Because participants were selected from household units, it is possible that refugees and irregular immigrants were under-represented. Second, this study is prone to recall bias due to the self-reported nature of the outcome variable. As a result, this could have led to an underestimation of the vaccination coverage [35,36]. Third, due to anonymity of data, Statistics Portugal did not provide participants’ country of birth, and for this reason we used the variable region of birth as coded by them. The category “Non-EU member states” is very broad and therefore our results for this variable need to be interpreted with caution regarding the lack of association with our outcome variable. Fourth, the information available in the NHS 2014 did not include migration-related factors that may play an important role in accessing healthcare services, such as language proficiency. For this reason, we were not able to undertake a more comprehensive analysis including all the variables that we consider important when studying tetanus vaccination coverage among adult immigrants in Portugal. Finally, data used in this study were collected in 2014, and the current situation may be different due to several factors, namely changes in the composition of the foreign population living in Portugal. These changes may impact the current self-reported tetanus vaccination coverage and its related factors. Nevertheless, to our knowledge, this was the first study to identify the factors associated with tetanus vaccination among adult immigrants in Portugal, and these results may be useful to compare with future studies.

## 5. Conclusions

This study identified several sociodemographic, migration-related, and access-to-care factors associated with self-reported tetanus vaccination coverage among adult immigrants in Portugal. Our findings showed that age, household income per adult, region of residence, citizenship, private health insurance, and contact with a family/general physician are associated with the outcome variable. Action to improve vaccination services for adult immigrants should consider the determinants found in this study, and address missed opportunities for vaccination.

## Figures and Tables

**Table 1:** Sociodemographic, migration-related, and access-to-care characteristics of adult immigrants (≥18 years of age) in Portugal, National Health Survey 2014.

Characteristics	n	Weighted %
**Overall**	1277	100.0
**Gender**		
Male	535	45.9
Female	742	54.1
**Age**, years (mean ± SD)	44 ± 14	
**Marital status**		
Married/live with partner	781	67.0
Widowed/Divorced	198	9.9
Never married	298	23.1
**Education level**		
No education/basic	491	39.0
Secondary	393	32.9
Higher	393	28.1
**Employment status**		
Employed	780	61.4
Unemployed	220	18.5
Other ^a^	277	20.1
**Household income per adult**		
Quintile 1	221	20.5
Quintile 2	209	16.2
Quintile 3	260	22.5
Quintile 4	244	16.2
Quintile 5	343	24.6
**Degree of urbanization**		
Densely populated area	458	57.1
Intermediate density area	479	24.1
Thinly populated area	340	18.8
**Region of residence**		
*Norte*	122	21.9
*Centro*	229	19.1
*Lisboa*	234	44.1
*Alentejo*	99	3.3
*Algarve*	374	8.3
*RA dos Açores*	69	1.0
*RA da Madeira*	150	2.3
**Self-perceived health status**		
Very good/Good	823	62.2
Fair/Poor/Very poor	454	37.8
**Region of birth**		
EU member states	371	24.4
Non-EU member states	906	75.6
**Length of residence**, years (median)	26	
**Citizenship**		
Portuguese citizen	887	74.7
Not Portuguese citizen	390	25.3
**Public healthcare**		
Only National Health Service	1049	81.6
National Health Service and health subsystems	228	18.4
**Private health insurance**		
No	946	75.4
Yes	331	24.6
**Last contact with family/general physician**		
≥12 months	436	29.6
<12 months	841	70.4

^a^ Other includes students, pensioners, permanently incapacitated, housewives, and other status. SD—standard deviation; EU—European Union; *RA*—*Região Autónoma* (Autonomous region).

**Table 2:** Determinants of self-reported tetanus vaccination coverage among adult immigrants (≥18 years of age) living in Portugal, National Health Survey 2014 ^§^.

Variables	Tetanus Vaccination Coverage % (95% CI)	aOR (95% CI)	*p* Value
**Gender**			
Male	82.7 (77.4; 87.0)	Ref.	
Female	76.9 (71.2; 81.7)	0.73 (0.47; 1.15)	0.179
**Age** (years)	-	0.97 (0.95; 0.99)	0.011
**Marital status**			
Married/live with partner	78.0 (73.0; 82.3)	Ref.	
Widowed/Divorced	74.5 (64.4; 82.6)	1.17 (0.62; 2.21)	0.628
Never married	86.1 (80.0; 90.6)	1.60 (0.88; 2.89)	0.123
**Education level**			
No education/basic	77.6 (71.1; 83.0)	Ref.	
Secondary	81.3 (74.9; 86.3)	1.03 (0.58; 1.84)	0.915
Higher	80.2 (72.9; 85.9)	0.94 (0.49; 1.79)	0.838
**Employment status**			
Employed	82.6 (77.9; 86.5)	Ref.	
Unemployed	76.8 (67.5; 84.1)	0.77 (0.42; 1.42)	0.401
Other ^a^	72.7 (62.8; 80.7)	0.89 (0.50; 1.58)	0.680
**Household income per adult**			
Quintile 1	77.5 (68.0; 84.8)	Ref.	
Quintile 2	78.9 (67.9; 86.8)	0.83 (0.39; 1.78)	0.633
Quintile 3	87.8 (81.2; 92.3)	1.35 (0.66; 2.79)	0.415
Quintile 4	78.8 (68.1; 86.7)	0.64 (0.29; 1.41)	0.268
Quintile 5	74.6 (67.2; 80.8)	0.42 (0.19; 0.96)	0.039
**Degree of urbanization**			
Densely populated area	75.2 (69.3; 80.3)	Ref.	
Intermediate density area	82.7 (76.7; 87.4)	1.03 (0.49; 2.18)	0.934
Thinly populated area	88.6 (83.4; 92.4)	1.32 (0.62; 2.81)	0.466
**Region of residence**			
*Norte*	90.4 (81.8; 95.2)	Ref.	
*Centro*	92.0 (86.8; 95.2)	1.22 (0.42; 3.56)	0.722
*Lisboa*	70.8 (63.7; 77.1)	0.28 (0.12; 0.64)	0.003
*Alentejo*	77.6 (69.0; 84.3)	0.40 (0.14; 1.16)	0.091
*Algarve*	69.4 (63.3; 74.8)	0.31 (0.11; 0.82)	0.019
*RA dos Açores*	85.2 (72.9; 92.5)	0.47 (0.15; 1.49)	0.200
*RA da Madeira*	76.5 (69.0; 82.7)	0.30 (0.11; 0.77)	0.013
**Self-perceived health status**			
Very good/Good	82.3 (77.9; 86.0)	1.48 (0.90; 2.43)	0.122
Fair/Poor/Very poor	75.0 (68.0; 80.9)	Ref.	
**Region of birth**			
EU member states	84.9 (78.2; 89.8)	Ref.	
Non-EU member states	77.8 (73.5; 81.6)	0.91 (0.49; 1.69)	0.775
**Length of residence** (years)	-	1.01 (0.99; 1.03)	0.504
**Citizenship**			
Portuguese citizen	83.1 (78.8; 86.7)	2.30 (1.25; 4.24)	0.008
Not Portuguese citizen	69.1 (61.8; 75.6)	Ref.	
**Public healthcare**			
Only National Health Service	79.9 (76.0; 83.3)	Ref.	
National Health Service and health subsystems	77.9 (67.8; 85.5)	1.47 (0.79; 2.76)	0.227
**Private health insurance**			
No	78.1 (73.5; 82.1)	Ref.	
Yes	84.0 (77.1; 89.2)	1.99 (1.06; 3.71)	0.032
**Last contact with family/general physician**			
≥12 months	76.7 (70.1; 82.3)	Ref.	
<12 months	80.7 (76.3; 84.5)	1.59 (1.01; 2.51)	0.046

^§^ All results shown in this table were obtained with SPSS complex samples module, which incorporates strata, primary sampling units, and weight variables. ^a^ Other includes students, pensioners, permanently incapacitated, housewives, and other status. aOR—Adjusted Odds Ratio; CI—Confidence Interval; EU—European Union; *RA*—*Região Autónoma* (Autonomous region); Ref.—Reference category.

## Supplementary Materials

The following are available online at https://www.mdpi.com/1660-4601/16/9/1619/s1, Table S1: Self-reported tetanus vaccination coverage by country of birth among adults (≥18 years of age) living in Portugal, National Health Survey 2014.
