# Supplementary material for: Determinants of Tetanus Vaccination among Adult Immigrants: Findings from the Portuguese National Health Survey 2014
Source: Int J Environ Res Public Health. 2019 May 9;16(9):1619. doi: 10.3390/ijerph16091619 (PMC6539381; doi:10.3390/ijerph16091619)
Supplement: Supplementary file 1 [file ijerph-16-01619-s001.pdf]

International Journal of
Environmental Research and Public Health

MDPI

Supplementary File 1

Table S1. Self-reported tetanus vaccination coverage by country of birth among adults (≥18 years of age) living in Portugal, National Health Survey 2014[§] (n=17,432).

	**n**	**Weighted %**	**Tetanus vaccination coverage % (95% CI)**	**aOR[a] (95% CI)**	**p value**
**Country of birth**					
Portugal	16,155	92.1	83.0 (81,9; 84.0)	Ref.	
Other	1277	7.9	79.5 (75.8; 82.8)	0.61 (0.49; 0.77)	<0.001

[§] All results shown in this table were obtained with SPSS complex samples module, which incorporates strata, primary sampling units, and weight variables. [a] Adjusted for sex, age, educational level, and household income per adult. aOR—Adjusted Odds Ratio; CI—Confidence Interval; Ref. —Reference category.
